# Efficacy of Automated Red Cell Exchange for Sickle Cell Disease: A Cross-Sectional Study

**DOI:** 10.7759/cureus.106500

**Published:** 2026-04-06

**Authors:** Piyush Kumar Sharma, Ramu Thakur, Amrita Tripathi, Karuna Thakur, Ashok Yadav, Saifaly Gupta

**Affiliations:** 1 Immunohematology and Blood Transfusion, Mahatma Gandhi Memorial Medical College, Indore, IND; 2 Medicine, Mahatma Gandhi Memorial Medical College, Indore, IND; 3 Community Medicine, Employees' State Insurance Corporation (ESIC), Indore, IND

**Keywords:** acute chest syndrome, automated red cell exchange, packed red blood cells, sickle cell disease, vaso-occlusive crisis

## Abstract

Background: Sickle cell disease is a hereditary hemoglobinopathy commonly seen in sub-Saharan Africa, India, and the Middle East. It is caused by a mutation in the β-globin gene and is associated with both acute and chronic multiorgan complications, including severe anemia and stroke.

Materials and methods: This cross-sectional study was carried out over a period of one year (2022-2023) among patients with sickle cell disease. Automated red cell exchange was performed using a continuous-flow cell separator to assess the procedural efficiency.

Results: A total of 30 male and female patients with sickle cell disease from districts surrounding Indore were included. The mean age was 23.4 years, and procedures were performed for various indications as per the American Society for Apheresis (ASFA) guidelines. Most cases, 27 (90%), fell under category II. Thirty automated red cell exchange procedures were performed. Among these, 17 (57%) patients achieved a hemoglobin S (HbS) reduction of 71-80%, eight (27%) >81% reduction, four (13%) 61-70% reduction, and one (3%) <60% reduction.

Conclusion: Automated red cell exchange led to significant improvement in multiple hematological parameters, including hemoglobin levels, hematocrit, indirect bilirubin, and HbS percentage, supporting its effectiveness in the management of sickle cell disease.

## Introduction

Sickle cell disease (SCD) is a qualitative type of hemoglobinopathy inherited as an autosomal recessive hematological disorder with acute and chronic complications. There are around 300,000 new cases globally each year [1]. It is also widespread among the tribal population in India, where about one in 86 births among the scheduled tribes has SCD. Madhya Pradesh is one of the high-prevalence states in India [2]. For the reduction in the prevalence of SCD, several national and state-specific initiatives have been planned and undertaken [3].

Hemoglobin is a tetrameric protein composed of two alpha and two beta globin chains. In SCD, a single nucleotide substitution at the sixth position of the beta-globin gene results in the replacement of negatively charged glutamic acid with neutral valine, producing hemoglobin S (HbS). Under deoxygenated conditions, HbS polymerizes, causing erythrocyte sickling and subsequent hemolysis, vaso-occlusion, and end-organ damage. This sickle hemoglobin becomes rigid and elongated and forms a polymer in a deoxygenated state. Initially, this process is reversible, but on repeated exposures, it becomes irreversible, causes acute or chronic complications, and is clinically presented as hemolytic anemia, fever, vaso-occlusive crisis, acute chest syndrome, stroke, and transient ischemic attack (TIA) [2,3].

Automated red cell exchange is an apheresis-based procedure that removes a proportion of the patient's red blood cells (including both sickled and normal erythrocytes) and replaces them with donor red blood cells, thereby reducing the HbS concentration without increasing the hematocrit level or causing iron overload. It also helps the patient to avoid repeated visits to the hospital, so the objective of this study is to evaluate HbS reduction and hematological parameter changes following automated red cell exchange.

## Materials and methods

This cross-sectional observational study was conducted over one year (2022-2023) at the Department of Immunohematology and Blood Transfusion, Maharaja Yeshwantrao Hospital, a tertiary care hospital in Indore, Madhya Pradesh, India. A total of 30 patients of all age groups with acute and chronic complications of SCD, categorized under the American Society for Apheresis (ASFA) guidelines (categories I, II, and III), were included. The sample size was determined based on feasibility and the availability of eligible cases during the study period, and all consecutive patients meeting the inclusion criteria and requiring automated red cell exchange were enrolled. Comparable sample sizes have been reported in similar studies, supporting the adequacy of this sample for the preliminary evaluation of efficacy outcomes. Efficacy was assessed based on the reduction in HbS% and changes in hematological parameters following the procedure.

Ethical approval

Ethical clearance was obtained from the Ethics and Scientific Review Committee of the Mahatma Gandhi Memorial Medical College and Maharaja Yeshwantrao Hospital under the approval number EC/MGM/DEC-23/21. The anonymity and confidentiality of the participants were guaranteed. Each participant was informed about the objective of the study, and informed written consent was taken from the legal guardian for those below 18 years of age or from the participant over 18 years.

Selection of participants

Each participant underwent a thorough pre-procedure evaluation, including vital sign assessment, determination of ASFA indication category, and evaluation of procedural tolerance. The decision to perform this procedure on SCD patients was based on clinical indications for red cell exchange (Table 1) following the ASFA guidelines [4]. The procedure was explained to the patient, and informed consent was obtained from all the patients.

**Table 1:** ASFA indication for red cell exchange ASFA: American Society for Apheresis

S. no.	Indication	ASFA category	Level of evidence
Sickle cell disease, acute			
1	Acute stroke	Category I	1C
2	Acute chest syndrome	Category II	1C
3	Other complications	Category III	2C
Sickle cell disease, non-acute			
1	Stroke prophylaxis	Category I	1A
2	Pregnancy	Category II	2B
3	Recurrent vaso-occlusive crisis	Category II	2B
4	Preoperative management	Category III	2A

Inclusion and exclusion criteria

All patients with SCD who are in sickle cell crisis and for automated red cell exchange as per the ASFA indication categories I, II, and III were included in the study. In contrast, excluded were patients who were non-compliant with the procedure, patients not consenting to participate in the study, and patients coming under category IV of the ASFA guideline.

Red cell unit selection

Selection of red cell concentrate (RCC) units used for exchange transfusion was carried out as per the departmental standard operating procedures. The red blood cell (RBC) units were prepared from 350 mL of whole blood collected in triple blood bags containing citrate phosphate dextrose (CPD)/saline adenine glucose mannitol (SAGM) or citrate-phosphate-dextrose-adenine (CPDA) anticoagulant-preservative solutions. Fresh, ABO- and Rh-compatible RBC units of the same blood group, collected within the preceding seven days, were selected for transfusion. All units were screened and found serologically negative by both enzyme-linked immunosorbent assay (ELISA) and nucleic acid amplification test (NAT). Leukoreduced units were used, with confirmation of the patient's ABO and Rh status by both forward and reverse grouping, along with antibody screening using the conventional tube technique and compatibility testing by anti-human globulin (AHG) crossmatch.

Vascular access

This is a dual vascular access procedure, so this machine requires a separate inlet and return peripheral line or dual line central venous catheter in either the internal jugular vein or femoral vein.

Procedure

**Figure 1:**
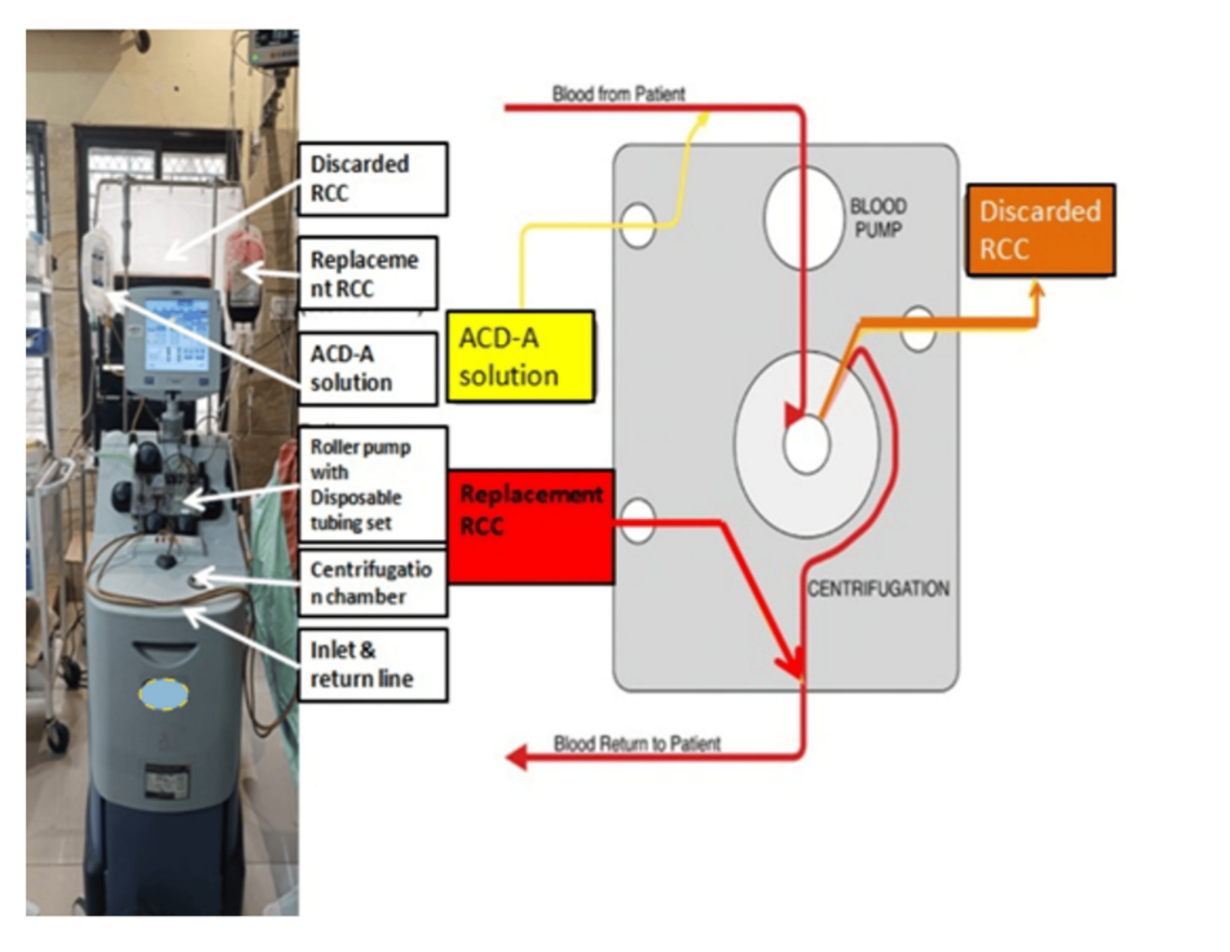
Schematic diagram showing the red cell exchange RCC: red cell concentrate; ACD-A: anticoagulant citrate dextrose solution A

All procedures were performed on the Spectra Optia continuous flow cell separator (Terumo Blood and Cell Technologies, Lakewood, Colorado, United States). Patients' parameters (sex, weight, height, hematocrit) and replacement fluid parameters (exchange fluid hematocrit) were entered into the machine, and the procedure was started. Calcium infusion was started during the procedure to manage the citrate toxicity. During the procedure, vital signs were recorded every 15 minutes. It takes 2-3 hours to complete the procedure (Figure 1).

Study analysis

Continuous data were expressed as mean±standard deviation, and categorical data were expressed as proportions and percentages. The paired t-test and the applied Wilcoxon signed-rank test were used for comparison of data. Statistical significance level was set at p=0.05. IBM SPSS Statistics for Windows, Version 25.0 (IBM Corp., Armonk, New York, United States), was used.

## Results

Thirty procedures were performed on 30 patients (20 male, 10 female) with SCD presenting with acute or chronic complications. All patients in the study were diagnosed with homozygous sickle cell disease (HbSS), as confirmed by hemoglobin electrophoresis. The study included a total of 30 participants from Indore and nine surrounding districts.

**Figure 2:**
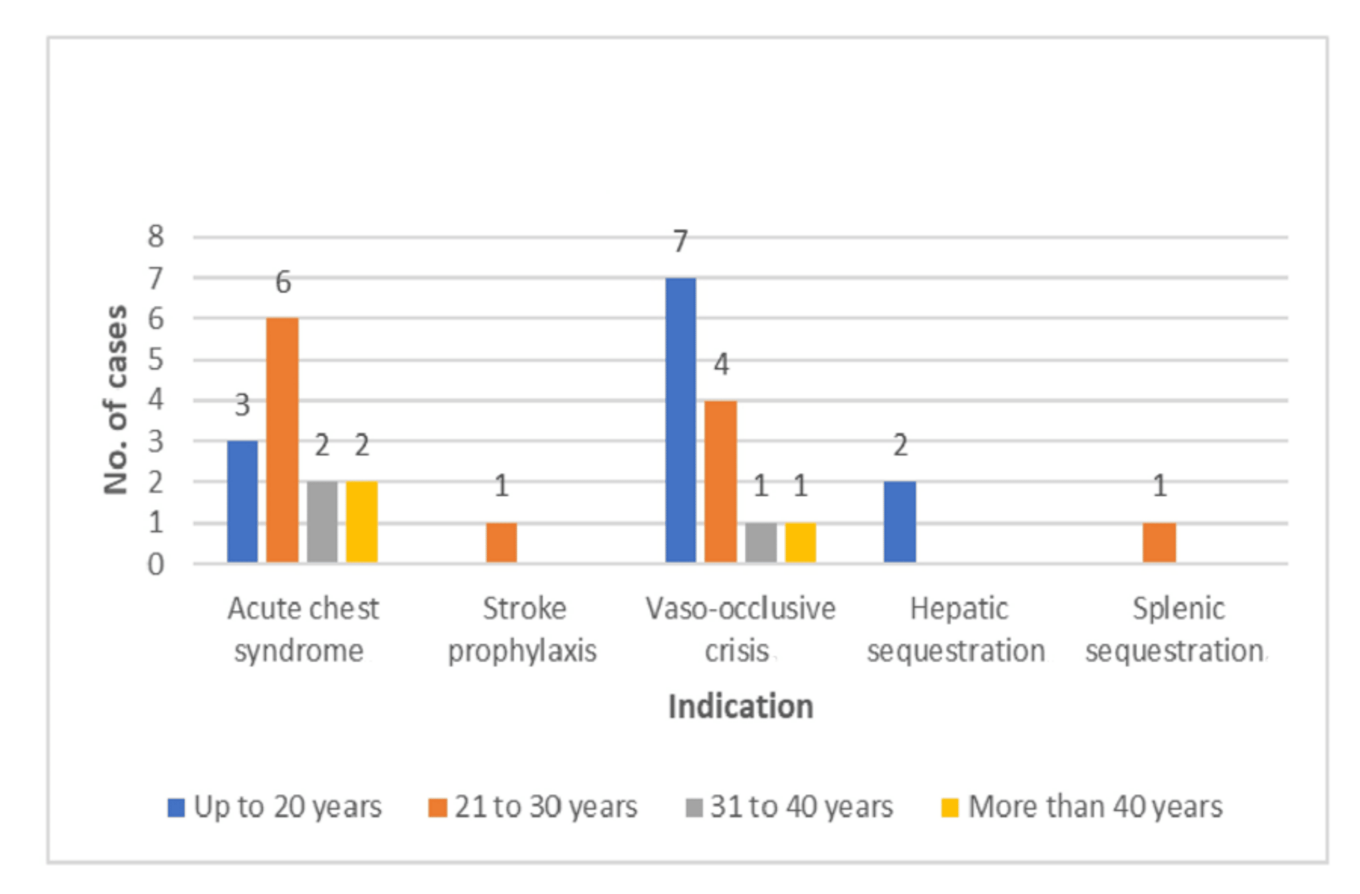
Distribution of case indications on the basis of age group

All 30 patients were admitted with different indications, like 13 (43.3%) with acute chest syndrome, 13 (43.3) with vaso-occlusive crisis, two (6.6%) with hepatic sequestration, and one (3.3%) each for splenic sequestration and stroke prophylaxis, respectively (Figure 2). The mean and range value of patients' age, weight, and height were 23.4 years (R=13-47), 44 kg (R=20-60), 153 cm (R=120-172), respectively (Table 2).

**Table 2:** Mean and range value of different parameters

Parameter	Mean	Range
Age (years)	23.4 (median: 24)	13-47
Weight (kg)	44	20-60
Height (cm)	153	120-172

**Figure 3:**
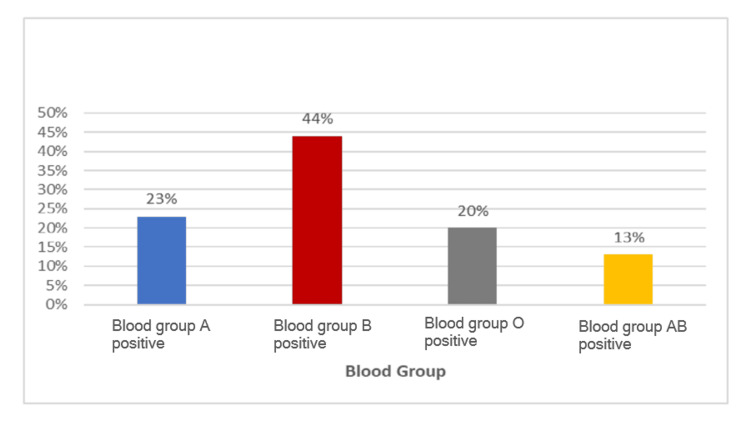
Blood group-wise distribution of cases

In the present study, out of all cases, 27 (90%) were in the ASFA category II (this is because most patients presented with indications falling under category II of the ASFA guidelines, such as recurrent vaso-occlusive crises and acute chest syndrome), one (3%) was in category I, and two (6%) were in category III, respectively (Table 3). All patients had different blood groups. Thirteen (44%) were of blood group B Rh D positive, seven (23%) blood group A Rh D positive, six (20%) blood group O Rh D positive, and four (13%) blood group AB Rh D positive (Figure 3). 

**Table 3:** Gender-wise distribution of patients as per the ASFA category ASFA: American Society for Apheresis

ASFA category	Male		Female	Total cases (%)
Category I	1		NIL	1 (3%)
Category II	18		9	27 (90%)
Category III	1		1	2 (6%)
Total		20	10	30

In the present study, out of 30 cases, 17 (57%) show HbS reduction between 71% and 80%, eight (27%) more than 81% HbS reduction, four (13%) 61-70% HbS reduction, and one (3%) HbS reduction of less than 60% (Table 4). Pre- and post-procedure comparisons are shown in Table 5, with median (range) reported for hematocrit, white blood cell count, platelet count, indirect bilirubin, HbS%, and serum calcium and mean (range) reported for hemoglobin.

**Table 4:** HbS% of patients HbS: hemoglobin S

HbS% reduction		No. of cases	Percentage
More than 81%		8	27%
71 -80%		17	57%
61-70%		4	13%
Less than 60%		1	3%
Total	30		100

**Table 5:** Pre- and post- procedure details of the hematological parameter of patients WBC: white blood cell; HbS: hemoglobin S

S. no.	Parameters	Pre-procedure		Post-procedure		P-value
		Median	Range	Median	Range	
1	Hematocrit (%)	25.5	20-34	28	24-30	0.002
2	WBC count (cells/mm^3^)	11710	4300-50370	10600	4100-24000	0.001
3	Platelet count (lakhs/mm^3^)	1.9	0.76-5.97	1.47	0.56-3.45	0.001
4	Indirect bilirubin (mg/dl)	5.75	0.24-20	1.8	0.2-19	0.001
5	HbS% (%)	72.5	55-90	17	9-51	0.001
6	Serum calcium (mg/dl)	8.65	7.3-9.9	8.7	7.8-9.5	0.2
7	Hemoglobin	8.4(±1.31) gm% (mean)	5.6-10.8	9.5(±0.99) gm% (mean)	7.6-11	0.001

## Discussion

The present study was conducted in a tertiary care hospital in Indore, Madhya Pradesh, India. Because this region lies within the sickle cell belt, there is a high prevalence of SCD among the tribal populations in Madhya Pradesh. Automated red cell exchange reduces the proportion of HbS-containing erythrocytes in the circulation, thereby decreasing the risk of sickling and providing symptomatic improvement in both acute and chronic SCD [5,6]. 

A continuous-flow cell separator (apheresis system) was used to perform the procedure. Similar apheresis systems have been used by Chowdhry et al. [5], Daniel et al. [6], Kanungo et al. [7], Tsitsikas et al. [8], Iyyapan et al. [9], Escobar et al. [10], Janes et al. [11], Kim et al. [12], Kalff et al. [13], and Hequet et al. [14] for automated red cell exchange procedures.

In the present study, a total of 30 patients were included, of which 12 (40%) were below 20 years of age, 12 (40%) between 21 and 30 years of age, and three (10%) each between 31 and 40 years and more than 40 years of age, respectively. The study population was predominantly young, with 24 (80%) patients being below 30 years of age. In the study by Kanungo et al. [7], out of nine patients, one was below 20 years of age, six were between 21 and 30 years of age, and two were between 31 and 40 years of age.

The male predominance observed in the present study (67%) is comparable to several previous studies, including Daniel et al. [6], Kanungo et al. [7], Janes et al. [11], Kim et al. [12], Kalff et al. [13], Al Mozain et al. [15], and Dedeken et al. [16], all of which also reported a higher proportion of male patients. In contrast, studies by Tsitsikas et al. [8], Hequet et al. [14], and Kozanoglu et al. [17] demonstrated a more balanced or female-predominant distribution. These variations in gender distribution across studies may be attributed to differences in referral patterns, regional disease prevalence, and sample sizes among different institutions (Table 6).

**Table 6:** Comparison of gender distribution with other studies

Study (author)	Sample size	Male (%)	Female (%)
Present study	30	20 (67%)	10 (33%)
Daniel et al. [6]	18	11 (69%)	7 (31%)
Kanungo et al. [7]	9	8 (89%)	1 (11%)
Tsitsikas et al. [8]	88	42 (48%)	46 (52%)
Janes et al. [11]	15	11 (73%)	4 (27%)
Kim et al. [12]	19	10 (53%)	9 (47%)
Kalff et al. [13]	13	10 (77%)	3 (23%)
Hequet et al. [14]	12	5 (42%)	7 (58%)
Al Mozain et al. [15]	11	10 (91%)	1 (9%)
Dedeken et al. [16]	10	8 (80%)	2 (20%)
Kozanoglu et al. [17]	83	35 (42%)	48 (58%)

In the present study, out of 30 cases, 13 (43%) had acute chest syndrome, 13 (43%) vaso-occlusive crisis, two (6%) hepatic sequestration, one (3%) acute stroke, and one (3%) had splenic sequestration. The two most common conditions were acute chest syndrome and vaso-occlusive crisis, each occurring in 13 (43%) cases. In all cases, the majority of patients, 27 (90%), were classified as ASFA category II, two (6%) were classified as ASFA category III, and one (3%) was classified as ASFA category I.

Across studies, the indications for red cell exchange vary, but vaso-occlusive crisis, secondary stroke prevention, and pre-surgical management are the most common. Most studies, including those by Daniel et al. [6], Aggarwal et al. [18], and Kanungo et al. [7], reported a predominance of ASFA category III indications, while Al Mozain et al. [15] and Tsitsikas et al. [8] showed a mix of categories II and III, with some category I cases in larger cohorts (Table 7).

**Table 7:** Comparison of indications and ASFA categories ASFA: American Society for Apheresis; VOC: vaso-occlusive crisis; ACS: acute chest syndrome

Study	Common indications	ASFA category distribution
Al Mozain et al. [15]	Secondary stroke prophylaxis (25%), VOC (25%), priapism (25%), ACS (15%)	II: 45%; III: 55%
Daniel et al. [6]	Pre-surgery management (100%)	III: 100%
Kanungo et al. [7]	VOC (44%), pre-surgery (33%), hepatopathy (22%)	III: 100%
Tsitsikas et al. [8]	VOC (most common), stroke prevention, leg ulcer, priapism, ACS	I: 15%; II: 64%; III: 21%
Aggarwal et al. [18]	Pre-surgery management	III: 100%

In the present study, the majority of patients had blood group B Rh D positive (44%), followed by A Rh D positive (23%), O Rh D positive (20%), and AB Rh D positive (13%). Similar to our findings, all patients in the studies by Al Mozain et al. [15], Kanungo et al. [7], and Choi et al. [19] were also Rh D positive. However, the distribution of ABO blood groups varied across studies, with O and A groups being more common in some studies, while B group predominance was observed in the present study (Table 8).

**Table 8:** Comparison of blood group distribution across studies

Study	Sample size	A+	B+	O+	AB+	Rh D status
Present study	30	7 (23%)	13 (44%)	6 (20%)	4 (13%)	All positive
Al Mozain et al. [15]	11	4 (36%)	2 (18%)	5 (46%)	0	All positive
Kanungo et al. [7]	9	2 (22%)	3 (33%)	3 (33%)	1 (11%)	All positive
Choi et al. [19]	3	0	2 (67%)	1 (33%)	0	All positive

In the present study, 57% of patients achieved HbS reduction of 71-80%, and 27% achieved >81%, indicating predominantly moderate to high reduction. Similar findings were reported by Kanungo et al. [7], while Aggarwal et al. [18] and Iyyapan et al. [9] observed moderate reductions (~60%). In contrast, Kalff et al. [13] reported lower reductions (<60%), whereas Choi et al. [19] demonstrated >80% reduction in all patients. These variations may be due to differences in clinical indications and target HbS levels as per the ASFA guidelines (Table 9).

**Table 9:** Comparison of HbS reduction (%) with other studies HbS: hemoglobin S

Study	HbS reduction (%)	Key observation
Present study	71-80% (57%), >81% (27%)	Majority achieved moderate to high reduction
Kanungo et al. [7]	Similar pattern	Comparable distribution
Aggarwal et al. [18]	~62%	Moderate reduction
Iyyapan et al. [9]	~60%	Moderate reduction
Kalff et al. [13]	Mostly <60%	Lower reduction in the majority
Choi et al. [19]	>80% (all patients)	Very high reduction

Automated red cell exchange in the present study led to improved hematological parameters, with a mild rise in hemoglobin and hematocrit and a marked reduction in HbS levels (73% to 17%). White blood cell counts decreased slightly, while serum calcium remained stable. Although significant post-procedure thrombocytopenia was observed, with a median reduction of 30% in platelet count, no patients had clinically significant bleeding complications, indicating procedural safety.

These findings are consistent with previous studies by Al Mozain et al. [15], Daniel et al. [6], Kanungo et al. [7], Tsitsikas et al. [8], Aggarwal et al. [18], Iyyapan et al. [9], Escobar et al. [10], Kim et al. [12], Kalff et al. [13], Kozanoglu et al. [17], and Choi et al. [19], supporting the effectiveness and safety of automated red cell exchange with consistent HbS reduction and transient platelet decline (Table 10).

**Table 10:** Comparison of hematological parameters with other studies Hb: hemoglobin; Hct: hematocrit; HbS: hemoglobin S

Study	Hb (pre → post)	Hct (pre → post)	Platelets (pre → post)	HbS% (pre → post)	Key finding
Present study	8.4 → 9.5	26 → 28	1.93 → 1.47 lakh	73 → 17	Significant HbS reduction; transient thrombocytopenia
Al Mozain et al. [15]	-	-	-	54.4 → 24.5	Marked HbS reduction
Daniel et al. [6]	-	-	-	73–85 → 22-29	Effective HbS lowering
Kanungo et al. [7]	10.1 → 11	30 → 32.4	2.18 → 1.88 lakh	67.4 → 30.7	Improvement in Hb/Hct; HbS reduction
Tsitsikas et al. [8]	-	26 → 30	3.57 → 1.40 lakh	44 → 12	Significant HbS drop; platelet reduction
Aggarwal et al. [18]	12.5 → 12.5	34.5 → 35	-	65.4 → 25.6	HbS reduction without Hb change
Iyyapan et al. [9]	9.5 → 10.1	27 → 31	-	74 → 18	Improved Hb/Hct; marked HbS drop
Escobar et al. [10]	8.4 → 8.6	23.5 → 23.8	3.93 → 2.56 lakh	66.3 → 35.8	Moderate HbS reduction
Kim et al. [12]	-	28.2 → 34.3	3.56 → 1.32 lakh	39.7 → 10.6	Significant HbS reduction
Kalff et al. [13]	-	-	-	47.3 → 25.4	Effective HbS reduction
Kozanoglu et al. [17]	-	24.3 → 27.5	4.13 → 3.49 lakh	74 → 27	HbS reduction with stable counts
Choi et al. [19]	-	28.2 → 29.2	-	74.8 → 3.9	Profound HbS reduction

Automated red cell exchange provided rapid clinical stabilization in acute complications like acute chest syndrome and vaso-occlusive crisis while minimizing the risk of iron overload. The importance of effective SCD management extends beyond acute symptom control. By reducing HbS levels and mitigating chronic hemolysis, regular red cell exchange may potentially reduce long-term cardiovascular and end-organ complications, though prospective studies are needed to confirm this benefit.

Limitations

The study has several limitations. First, its single-center design and relatively small sample size (n=30) restrict the generalizability of the findings. Second, the lack of a control group (such as patients receiving simple transfusion or standard care) limits the ability to compare efficacy. Third, the absence of long-term follow-up hinders the assessment of sustained clinical outcomes. Fourth, the possibility of selection bias cannot be excluded, as procedures were performed based on clinician discretion rather than randomization.

Furthermore, due to time and resource constraints, only a limited number of procedures could be carried out, which restricts a comprehensive evaluation of their benefits and drawbacks. In addition, some procedures were delayed or cancelled owing to challenges in arranging transfusions for patients with rare blood groups.

## Conclusions

This study demonstrates the efficacy and safety of automated red cell exchange in reducing HbS levels and improving hematological parameters in patients with SCD in a high-prevalence region. The procedure demonstrated a significant reduction in HbS levels in the majority of patients, with more than three-fourths achieving a reduction greater than 70%. Substantial improvement was also observed in key hematological parameters such as hemoglobin concentration, hematocrit, indirect bilirubin, and white blood cell counts following the procedure.

Automated red cell exchange provided rapid clinical stabilization in acute complications like acute chest syndrome and vaso-occlusive crisis while minimizing the risk of iron overload. Although transient thrombocytopenia was noted, no major bleeding or procedure-related adverse events occurred, indicating good procedural safety. The predominance of patients in ASFA category II reflects appropriate patient selection and adherence to established guidelines.

Overall, automated red cell exchange emerges as an effective therapeutic modality for both the acute management and prevention of long-term complications in SCD. Larger multicentric studies with long-term follow-up are recommended to further validate these findings and strengthen clinical practice guidelines.
